# Social Media Health Information Formats and Endometriosis Treatment-Seeking Intentions: A Randomized Controlled Trial

**DOI:** 10.1177/0272989X261436847

**Published:** 2026-04-13

**Authors:** Alice Graham, Brooke Nickel, Kirsten McCaffery, Jenny Doust, Erin Cvejic, Tessa Copp

**Affiliations:** Sydney Health Literacy Lab, Faculty of Medicine and Health, Sydney School of Public Health, The University of Sydney, Sydney, NSW, Australia; Sydney Health Literacy Lab, Faculty of Medicine and Health, Sydney School of Public Health, The University of Sydney, Sydney, NSW, Australia; Sydney Health Literacy Lab, Faculty of Medicine and Health, Sydney School of Public Health, The University of Sydney, Sydney, NSW, Australia; Australian Women and Girls’ Health Research Centre, School of Public Health, The University of Queensland, Saint Lucia, QLD, Australia; Sydney Health Literacy Lab, Faculty of Medicine and Health, Sydney School of Public Health, The University of Sydney, Sydney, NSW, Australia; Sydney Health Literacy Lab, Faculty of Medicine and Health, Sydney School of Public Health, The University of Sydney, Sydney, NSW, Australia

**Keywords:** Endometriosis, social media, laparoscopy, health communication

## Abstract

**Background:**

Despite many women learning about endometriosis on social media, posts about the condition often fail to reflect current evidence. With the content and credibility of online health messages being found to influence behavioral intentions in other areas, this study aimed to explore how the format of endometriosis-related social media posts affects women’s intentions to get a laparoscopy for the diagnosis and treatment of endometriosis.

**Design:**

In this 2 × 2 × (2) online randomized controlled trial, Australian women aged 18 to 45 y who had no prior endometriosis diagnosis were randomly assigned to view 1 of 4 mock Instagram posts. Post content (personal anecdote v. nonnarrative, factual information) and source (high-credibility “World Health Organization” (WHO) account v. low credibility layperson account) varied across conditions. A within-subjects component investigated change in intention when participants were informed of new treatment guidelines.

**Results:**

A total of 1,473 women were included in the analysis. Instagram posts featuring an anecdote produced higher treatment-seeking intentions (mean difference [MD] = 0.22, 95% confidence interval [CI] = 0.04–0.39) and more favorable attitudes toward getting a laparoscopy compared with posts containing nonnarrative information (MD = 0.13, 95% CI = 0.01–0.25). While the WHO account was perceived as more credible (MD = 0.29, 95% CI = 0.17–0.41), there were no differences in intentions, perceived norms, or self-efficacy toward laparoscopy compared with the layperson account. Advising participants of new evidence regarding the limitations of laparoscopy reduced intentions to get the procedure (MD = 0.29, 95% CI = 0.21–0.37), irrespective of condition.

**Conclusions:**

Findings demonstrate the power of anecdotes in shaping treatment preferences. Supplementing evidence-based information with personal anecdotes may ensure accurate yet engaging health information is used by online endometriosis communities to seek appropriate care. **Trial registration.** Australian New Zealand Clinical Trials Registry (https://www.anzctr.org.au/; ACTRN12624000767505p)

**Highlights:**

- Framing health information on Instagram as a personal anecdote increased women’s intentions to get a laparoscopy for the diagnosis and treatment of endometriosis.
- We found few differences between high- and low-credibility sources.
- Informing participants of new evidence and clinical guidelines reduced intentions across all conditions, potentially reflecting more informed decision making.

*In this study, we use the term “women” to reflect the population most affected by endometriosis. However, we acknowledge that individuals with diverse gender identities may also experience this condition. We use the terms “woman” and “women” throughout this article in the sexed sense of the words and not to exclude those who identify their gender differently. We aim to contribute to the understanding of the experiences of all those affected by endometriosis.*

Endometriosis is a chronic inflammatory condition that affects 1 in 10 women globally^1^ and occurs when tissue resembling the uterus lining grows outside of the womb.^2,3^ The growth of this excess scar tissue, known as lesions, can cause abdominal pain, heavy or irregular menstrual bleeding, constipation, diarrhea, and fatigue.^2,3^ However, the association between visible endometrial lesions and symptom severity is poorly understood, with some women experiencing no symptoms despite having severe lesions and others suffering from severe symptoms despite having only mild lesions.^4^ This varied symptom profile combined with the social normalization of period pain^5,6^ contribute to women experiencing an average of 4 to 9 y between symptom onset and receiving a diagnosis.^2,3,7^

Although pelvic exams and ultrasounds can suggest the presence of endometriosis, laparoscopy has long been considered the “gold standard” diagnostic method.^2^ This is a minimally invasive procedure in which endometrial lesions are identified and treated through surgical removal if found.^2,8^ However, laparoscopy is expensive,^9,10^ often requires long wait times,^11^ involves surgical risks,^12^ and symptom recurrence can be as high as 50% after 5 y.^8^ New evidence has also questioned whether laparoscopic surgical removal is more effective than pain medication and hormonal treatments, such as the oral contraceptive pill, in addressing symptoms^12–14^ and suggests the required surgical diagnosis may delay access to treatment.^11^

Recently published clinical guidelines in Australia^15^ and Europe^10^ have since advocated for a reevaluation of laparoscopy as the primary diagnosis and treatment approach, recommending it only in cases in which pain medication and hormonal treatments have failed to alleviate symptoms. Despite this new evidence, clinical practice and academic literature continue to prioritize surgical approaches, with laparoscopy often deemed the cornerstone of endometriosis management.^16^ Similar trends are seen on social media, with many posts about endometriosis maintaining that laparoscopy is the only accurate diagnostic method.^2^ Given that women with symptoms indicating possible endometriosis are increasingly turning to social media for immediate information,^3^ understanding how these posts influence women’s treatment-seeking behaviors has become particularly important.

People are increasingly seeking accessible health information on social media,^7^ which has coincided with the rise of the direct-to-consumer medical testing industry. Companies and social media influencers now use narratives of empowerment to promote medical screening tests as necessary, despite mixed evidence of their utility.^17^ This is of particular concern as, unlike other sources of health messaging, social media requires audiences to make their own judgments about the quality of the information and the credibility of the source.^18^ Within the context of endometriosis, a survey of 287 women found that more than 80% reported using social media to learn how to manage the condition.^3^ Yet, social media posts about endometriosis feature varying degrees of evidence-based information,^2,7^ and people with endometriosis report differing opinions on which social media sources they consider to be reliable.^3^

Prior literature suggests that when making health decisions, personal anecdotes are often more influential than nonnarrative information^19–21^ as they can shape beliefs about treatment efficacy,^19^ stimulate story-consistent attitudinal changes toward public health messages,^20^ and influence hypothetical treatment choices.^22,23^ In addition to message content, the perceived credibility of the source plays a critical role. Social media users perceive professional accounts as more credible than accounts of strangers or peers^24^ and are more likely to act on health information they provide.^25,26^ However, it remains possible that providing evidence-based information may counteract these persuasive effects. For example, informing women about the risk of overdetection in mammography screening^27^ and the unreliability of ultrasounds in diagnosing polycystic ovary syndrome (PCOS)^28^ has been shown to prompt a reconsideration of screening intentions.

Despite the content^19,20,29^ and credibility^25^ of health messages influencing behavioral intentions in other areas, the impact of different social media health message formats within the context of endometriosis remains unclear. This study aimed to test whether these effects also occur when communicating information about endometriosis by exploring how the content (personal anecdote v. nonnarrative) and source (high-credibility account v. low-credibility account) of endometriosis-related Instagram posts influence women’s intention to get a laparoscopy. We hypothesized that participants randomized to view the personal anecdote post would have higher intentions to get a laparoscopy and more favorable attitudes toward the procedure than those who viewed the nonnarrative, factual content. We also hypothesized that participants who viewed the post from a high-credibility source would report higher intentions to get a laparoscopy than those who viewed the same post from a low-credibility source. Consistent with the Integrative Model of Behavioural Prediction,^30^ which posits that behavioral intentions are shaped by attitudes, perceived norms, and self-efficacy, secondary outcomes explored whether these cognitive factors influence women’s intentions.

Further, given the recent shift away from laparoscopy in international guidelines due to uncertain evidence of benefit,^10,15^ this study also aimed to investigate whether intentions change when informed of new guidelines recommending laparoscopy only when pain medication and hormonal treatments fail to provide symptom relief. We hypothesized that providing this information will reduce intentions to undergo the procedure across all groups.

## Method

### Study Design

A 2 × 2 × (2) factorial design was used in which participants were randomly assigned to 1 of 4 conditions in which Instagram post content (personal anecdote v. nonnarrative factual information) and source credibility (high v. low) varied. A within-subjects component investigated the change in intention after presenting all participants with the same information about new endometriosis treatment guidelines. Ethics approval was granted by the University of Sydney Human Research Ethics Committee (2024/HE000624), and the study was prospectively registered with the Australian and New Zealand Clinical Trial Registry (ACTRN12624000767505p).

### Participants and Recruitment

Participants were recruited through the online social research company Dynata between June 27 and July 10, 2024. Dynata partners with multiple panels to recruit individuals who have preregistered to receive surveys. They randomly select participants based on target demographic criteria and use an incentivization system for participation in which rewards vary (e.g., cash, airline miles, gift cards). Women living in Australia who were 18 to 45 y of age and had not been diagnosed with endometriosis were eligible to take part in the study. Participation was voluntary and anonymous.

### Randomization and Allocation Concealment

Participants were randomly allocated to 1 of 4 conditions through Qualtrics survey software (Qualtrics randomizer feature) using a balanced allocation ratio. Using Web-based software, allocation and sequence generation were conducted automatically with participants and researchers blinded to the randomized condition.

### Intervention

Public Instagram posts about endometriosis were reviewed by the study authors for content and style. Stimuli were created to resemble Instagram posts using a mock-up template and free stock images, with their design being additionally informed by our extensive program of content analysis research examining online women’s health communication.^17,31^ For the post content study factor, participants were randomized to view a post that used either nonnarrative, factual information to describe endometriosis or a personal anecdote of the lived experience of having endometriosis. Both consisted of 5 image slides and a text caption outlining the prevalence, symptoms, and management of endometriosis. For the source credibility factor, participants were randomized to view a post from either a high- or low-credibility account. The high-credibility condition featured the World Health Organization (WHO) due to its position as a globally recognized public health authority.^32^ The low-credibility condition featured a layperson with the username “anonymous_user123” chosen to minimize perceived expertise, credentials, and affiliations. All posts featured the same background image, font, and number of “likes,” but the blue verification badge was included for the high-source-credibility condition (see Appendix A).

### Procedure

The online survey was created using Qualtrics. After indicating their informed consent, participants were asked about their use of social media for health-related purposes, their prior health care experiences, and demographic questions. They were then randomly allocated to view one of the Instagram posts. Following this, all participants were presented with the same hypothetical scenario (Box 1) asking them to imagine they had experienced common endometriosis symptoms for the past 12 mo and were consulting their general practitioner (GP) for diagnosis and treatment options. This reflects the typical care pathway for women with potential endometriosis symptoms in Australia, where they must first see a GP before being referred to specialist gynecologic care.^15^ They then completed the outcome measures. After this, all participants were presented with a second scenario (Box 2) asking them to imagine that their GP informed them of new evidence regarding the limitations of laparoscopy. Following this, participants were asked about their intention to undergo a laparoscopy again to assess any change.

**Box 1:** First Hypothetical Scenario of the General Practitioner (GP) Visit

Imagine that for the past 12 mo, you have had very painful periods. Sometimes, the pain is so intense that you have to call in sick to work. You also feel pain when you go to the bathroom and during sex. Worried that this is not normal, you make a visit to see your GP. After checking your symptoms and medical history, the doctor suggests you may have endometriosis. This is where uterine tissue grows outside of the womb. They explain that symptoms vary from person to person. You get an ultrasound scan, but the results are unclear. To help with your pain, the doctor suggests taking pain medicine. They also offer to refer you to a gynecologist, a doctor who specializes in a procedure called laparoscopy. The doctor explains this would involve having a general anesthetic (putting you to sleep) while a small cut is made in your stomach. The surgeon would then use a tiny camera to look for excess uterine tissue growing outside of the womb. If found, they will treat it by removing the tissue and send off a sample to confirm the diagnosis. They say that although this is the only way to diagnose endometriosis, the excess tissue can grow back. Also, improvement in pain may be only short-term. They also warn that long wait times are common, and it can cost between $500 and $4,000. Your doctor then asks, would you like to be referred to a gynecologist for laparoscopy?

**Box 2:** Second Hypothetical Scenario Outlining New Treatment Guidelines

Before you leave the appointment, the general practitioner remembers there are new guidelines on endometriosis. After reading the new guidelines, she says that laparoscopy is now recommended in only patients in whom pain medicine or hormonal treatment don’t work. She also said there is no evidence that an early diagnosis is better than a late diagnosis in preventing the progression of symptoms.

The evidence referred to in the nonnarrative post conditions and hypothetical scenarios was based on international clinical practice guidelines.^10,33^ The Instagram posts and hypothetical scenarios were developed and reviewed by a multidisciplinary team with expertise in psychology, public health, and women’s health, as well as a practicing GP with expertise in women’s health, to ensure accuracy and clinical relevance. To mirror real-world health decision making, in which individuals often first learn about a condition online before seeking diagnosis or treatment from a health care professional, laparoscopy was mentioned in only the hypothetical scenario and not in the Instagram posts. The questionnaire was first piloted with a convenience sample of 20 participants to ensure acceptability and comprehension. Instructions were revised to improve clarity, and the format of the hypothetical scenarios was edited to enhance readability. All other elements remained the same for the main study. No issues with the survey flow or randomization process were identified.

### Outcome Measures

A single item assessed hypothetical intention to get a laparoscopy for the diagnosis and treatment of endometriosis on a 7-point scale^34^ (“Which best describes your intentions to get a laparoscopy for the diagnosis and surgical treatment of endometriosis, answering as you would in the scenario above?,” 1 = *Definitely will not* to 7 = *Definitely will*; see Appendix B for full questionnaire) and was presented immediately after the first scenario (time 1) and after the second scenario (time 2). A free-text question followed asking participants to explain their choice. Secondary outcomes, including attitudes toward laparoscopy, perceived norms, self-efficacy, knowledge of endometriosis, psychosocial outcomes, and perceived source credibility, were assessed using various adapted, previously validated, and study-specific items designed to assess participants’ understanding of the information presented in the posts and scenario (Table 1).

**Table 1:** Secondary Outcome Measures

Variable	Description
Berlin Emotional Responses to Risk Instrument (BERRI)^35^	Six items on a 7-point scale. Scale scores were created by averaging scores across the measures of negative affect (BERRI-neg) and the measures of positive affect (BERRI-pos), with higher scores indicating a stronger emotional reaction to risk information.
Symptom worry^36^	“How worried would you feel about your symptoms described in the scenario above if you had them?” (4-point scale: 1 = *Not at all worried* to 4 = *Very worried*).
Attitudes toward laparoscopy^37^	Four items on a 7-point scale (1 = *Not at all* to 7 = *Extremely*). Relevant items were reverse coded, and items were averaged to create a composite score. Higher scores indicated a more favorable attitude toward laparoscopy.
Perceived norms toward laparoscopy^37^	Three items on a 7-point scale (1 = *Strongly disagree* to 7 = *Strongly agree*). Items were averaged to create a composite score, with higher scores indicating a stronger perception of normative support for laparoscopy.
Self-efficacy toward getting a laparoscopy^38^	“How confident are you that you can get a laparoscopy for the diagnosis of endometriosis?” (10-point scale: 1 = *Not at all confident* to 10 = *Completely confident*).
Perceived source credibility^25^	Six items on a 7-point scale (e.g., “The Instagram account sharing information about endometriosis is:” 1 = *Untrustworthy* to 7 = *Trustworthy*). Items were averaged to create a composite score, with higher scores indicating higher perceived credibility.
Knowledge of endometriosis	Ten items assessing participants’ understanding of endometriosis based on information presented in both the social media post and the scenario (e.g., “How many women are affected by endometriosis?” 0 = *Incorrect* or 1 = *Correct*). An overall score was computed by summing the items, with higher scores reflecting greater knowledge.

### Statistical Analysis

An a priori power analysis indicated a sample size of *N* = 800 (*n* = 200 per study arm) would be sufficient to detect main effects of content, source, and their interaction, with 90% power at an alpha of 0.05. The analysis assumed an expected effect size of Cohen’s *d* = 0.23. A soft launch of 214 participants was initially conducted to confirm sample size calculation assumptions. Soft launch results identified there would be sufficient power to detect the main effects of source credibility; however, due to greater variability than expected, a revised sample size of *N* = 1,448 (n = 362 per condition) would be needed to achieve 80% power to detect the main effects of post content.

Statistical Package for the Social Sciences (SPSS) version 29 was used with an alpha level of 0.05 set for all statistical tests. Prior to the analyses, the data were cleaned and checked for missing values, outliers, and nonserious responders. Relevant items were reverse scored, and composite scores were computed for scales with more than 1 item. Reliability analyses were conducted for the composite scales, with all showing high internal reliability in the current study. Descriptive statistics were used to characterize the sample. The differences between groups were analyzed using a 2 × 2 between-subjects analysis of variance (ANOVA) for primary and secondary outcomes. A repeated-measures mixed ANOVA was conducted to examine the change in intention. No adjustments were made for multiple comparisons.

### Content Analysis

An inductive content analysis was used for the free-text question asking participants to explain their intention to get a laparoscopy, with common themes extracted to develop a coding framework. Two researchers independently reviewed the free-text responses, and each developed an initial list of emerging codes and themes. The lists were then combined to create a coding framework, before being reviewed by a third researcher. To ensure consistency, reliability, and rigor, the first 200 were then double coded. The level of agreement was tested using Cohen’s kappa^39^ and indicated a moderate level of agreement (κ = 0.76). All remaining free-text responses were then coded into the framework, with descriptive statistical analysis being used to calculate the frequency of each code and exemplar quotes selected to illustrate findings. As participant responses often contained several distinct ideas, individual responses could receive multiple codes where relevant.

### Public and Patient Involvement Statement

While we recognize the importance of patient and public involvement in research, the design, recruitment, reporting, and dissemination plans for this study were executed without formal consultation with patient or public representatives. We will consider their input in future studies to enhance the impact and relevance of our findings.

## Results

**Figure 1:**
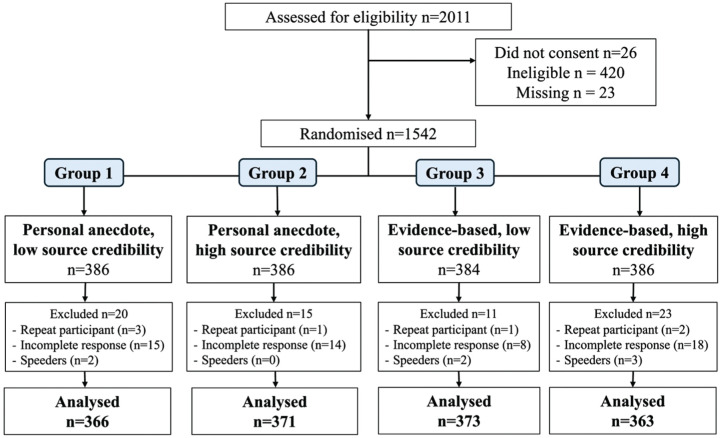
Participant flow diagram.

Of the 1,542 eligible participants who were randomized, 69 were excluded from the analysis due to having incomplete responses (*n* = 55), taking the survey more than once (*n* = 7), and completing the questionnaire at a speed less than one-third of the median duration indicating an inattentive or nonserious response (*n* = 7). This resulted in a total of 1,473 participants for the final analysis (Figure 1).

The demographic characteristics of the sample by condition are displayed in Table 2. The average age of the sample was 33.26 y (SD = 7.26, range = 18–45 y), and almost half of the sample had obtained a university degree or higher (46.3%, *n* = 682). Participants varied in the frequency of the use of social media for their health, and more than half (51.7%, *n* = 762) had seen a doctor for painful periods.

**Table 2:** Demographics by Randomized Condition

	Conditions 1 to 4							
	1. Personal Anecdote, Low Credibility		2. Personal Anecdote, High Credibility		3. Nonnarrative, Low Credibility		4. Nonnarrative, High Credibility	
Demographic Variable	M	SD	M	SD	M	SD	M	SD
Age (y)	33.66	7.26	33.41	6.95	32.72	7.48	33.28	7.32
	*n*	%	*n*	%	*n*	%	*n*	%
Highest level of education								
Less than year 12 or equivalent	31	8.5	23	6.2	10	2.7	40	11.0
Completed year 12 or equivalent	54	14.8	69	18.6	70	18.8	78	21.5
Trade, technical certificate, or diploma	108	29.5	96	25.9	121	32.4	91	25.1
University degree	132	36.1	131	35.3	122	32.7	112	30.9
Postgraduate/higher degree	41	11.2	52	14.0	50	13.4	42	11.6
Language at home								
English	337	92.1	337	90.8	351	94.1	338	93.1
Other	29	7.9	34	9.2	22	5.9	25	6.9
How well do you speak English?								
Very well	321	87.7	319	86.0	340	91.2	322	88.7
Well	42	11.5	50	13.5	30	8.0	41	11.3
Not well	3	0.8	2	0.5	3	0.8	0	0.0
Aboriginal or Torres Strait Islander origin								
Yes	28	7.7	29	7.8	24	6.4	29	8.0
No	334	91.3	340	91.6	348	93.3	331	91.2
Prefer not to say	4	1.1	2	0.5	1	0.3	3	0.8
Previously diagnosed health conditions								
Polycystic ovary syndrome	52	14.2	63	17.0	42	11.3	59	16.3
Uterine fibroids	14	3.8	20	5.4	11	2.9	12	3.3
Adenomyosis	7	1.9	4	1.1	10	2.7	5	1.4
Cancer	10	2.7	10	2.7	4	1.1	10	2.8
None	283	77.3	274	73.9	306	82.0	277	76.3
Seen a doctor for painful periods								
Yes	188	51.4	201	54.2	182	48.8	191	52.6
No	178	48.6	170	45.8	191	51.2	172	47.4
Frequency of social media for health								
Daily	73	19.9	61	16.4	66	17.7	68	18.7
Once a week	73	19.9	91	24.5	88	23.6	82	22.6
Once a month	67	18.3	70	18.9	62	16.6	75	20.7
Less than once a month	61	16.7	64	17.3	62	16.6	55	15.2
Never	92	25.1	85	22.9	95	25.3	83	22.9
Difficulties communicating symptoms to health professional								
Yes	207	56.6	199	53.6	219	58.7	226	62.3
No	159	43.4	172	46.4	154	41.3	137	37.7
Disbelieved by health professional								
Yes	214	58.5	208	56.1	206	55.2	217	59.8
No	152	41.5	163	43.9	167	44.8	146	40.2
Medical Minimizer Maximizer Scale								
Lean toward waiting and seeing	224	61.3	231	62.2	246	66.0	237	65.3
Lean toward taking action	142	38.9	140	37.7	127	34.1	126	34.7

### Intention to get a Laparoscopy

Table 3 shows outcome means by condition. Controlling for source credibility, intention to get a laparoscopy for the diagnosis and treatment of endometriosis was significantly higher for those who viewed the Instagram post featuring a personal anecdote than those who viewed the post containing nonnarrative content: mean difference (MD) = 0.22, 95% confidence interval (CI) = 0.04 to 0.39, *F*(1, 1,469) = 5.99, *P* = 0.015, $\eta_{p}^{2}=\;0.004$ . Controlling for post content, there was no significant difference in intention between those who viewed the Instagram post from the low or high credibility source: MD = 0.08, 95% CI = −0.10 to 0.25, *F*(1, 1,469) = 0.72, *P* = 0.40, $\eta_{p}^{2}=\;0.000$ . The interaction was also not significant, *F*(1, 1,469) = 0.22, *P* = 0.64, $\eta_{p}^{2}=\;0.000$ .

**Table 3:** Outcome Means by Condition

	Conditions 1 to 4							
Outcome Measures	1. Personal Anecdote, Low Credibility (*n* = 366)		2. Personal Anecdote, High Credibility (*n* = 371)		3. Nonnarrative, Low Credibility (*n* = 373)		4. Nonnarrative, High Credibility (*n* =363)	
	M	SD	M	SD	M	SD	M	SD
Intention (time 1)^a^	4.72	1.73	4.75	1.65	4.46	1.75	4.58	1.71
Intention (time 2)^b^	4.37	1.78	4.41	1.63	4.36	1.70	4.23	1.73
Attitudes	4.73	1.12	4.75	1.11	4.58	1.16	4.65	1.16
Beneficial	4.99	1.57	5.05	1.50	4.82	1.53	4.80	1.54
Harmful	4.19	1.49	4.09	1.57	4.08	1.48	4.09	1.57
Useful	5.02	1.44	5.08	1.39	4.79	1.48	5.02	1.49
Necessary	4.73	1.49	4.79	1.51	4.62	1.54	4.71	1.59
Perceived norms	5.07	1.23	5.08	1.22	4.98	1.16	5.02	1.26
Self-efficacy	5.99	2.42	6.20	2.36	6.01	2.50	6.08	2.51
Perceived source credibility	4.95	1.19	5.22	1.13	4.81	1.19	5.14	1.19
BERRI (positive)	5.04	1.39	5.00	1.45	4.68	1.56	4.71	1.48
Assured	5.07	1.24	5.01	1.36	4.76	1.52	4.82	1.40
Hopeful	5.25	2.08	5.24	2.05	4.85	2.17	4.82	2.15
Relieved	4.80	1.41	4.77	1.48	4.44	1.54	4.48	1.52
BERRI (negative)	4.04	1.54	4.10	1.62	4.21	1.47	4.42	1.40
Anxious	3.76	1.61	3.80	1.75	3.76	1.60	3.42	1.55
Afraid	4.11	1.72	4.03	1.76	3.88	1.65	3.74	1.54
Worried	4.01	1.71	3.86	1.72	3.73	1.60	3.58	1.53
Symptom worry	2.82	0.84	2.93	0.81	2.83	0.86	2.87	0.77
Knowledge	6.90	2.38	6.62	2.50	6.71	2.39	6.46	2.53

BERRI, Berlin Emotional Responses to Risk Instrument.

a Initial intention to get a laparoscopy.

b Intention to get a laparoscopy after being informed of new diagnosis and treatment guidelines.

When participants were asked to explain their intention (free-text answer), the top 3 codes from the content analysis were the same for those shown the personal anecdote and those shown the nonnarrative content: 1) it is too expensive (18.6%, *n* = 137 and 19.3%, *n* = 142, respectively), 2) to get a definitive diagnosis (18.2%, *n* = 134 and 16.6%, *n* = 122, respectively), or 3) to reduce pain and improve quality of life (13.6%, *n* = 100 and 11.4%, *n* = 84, respectively). See Table 4 for all codes and illustrative quotes.

**Table 4:** Content Analysis Results for the Free-Text Question regarding Intention (Time 1), “Which Best Describes Your Intentions to Get a Laparoscopy for Diagnosis and Surgical Treatment of Endometriosis, Answering as You Would in the Scenario Above? Please Tell Us Why”

Code	Example Quote	*n* (%)
Positive intention		
To get a definitive diagnosis/peace of mind	“I would want to be sure what’s going on.”	256 (17.4%)
To reduce pain/improve quality of life/daily functioning	“If I was in a lot of pain and it was impacting my life, I would want a resolution.”	184 (12.5%)
I think it’s important/I take my health seriously	“Health is extremely important.”	113 (7.7%)
To guide treatment/make informed treatment decisions	“If it means a step towards getting the right treatment then it is worth doing.”	70 (4.8%)
Trust in doctor	“I trust in health care professionals and their opinions.”	28 (1.9%)
Benefits outweigh the risks and costs	“It seems worthwhile for the amount of pain experienced, even if it wasn’t permanent.”	28 (1.9%)
To stop symptoms or condition from getting worse	“I would highly consider it if it meant it would reduce chances of long-term issues.”	27 (1.8%)
To maintain fertility	“Would consider it as I wouldn’t want it to impact my fertility.”	12 (0.8%)
Personal experience where it was effective	“I have previously had one to try and investigate my period pain.”	10 (0.7%)
To avoid long-term use of pain medication	“Better than nothing or relying on pain medication forever.”	7 (0.5%)
Negative intention		
Cost/too expensive	“I can’t afford the cost.”	279 (18.9%)
Temporary benefit/not effective/not worth it	“It’s only a short-term fix.”	128 (8.7%)
Fear/risks of surgery/too invasive	“I don’t want to have such an invasive procedure.”	122 (8.3%)
Would want to seek more information/second opinion	“I would need more information before committing to surgery.”	63 (4.3%)
I don’t think it’s needed/not relevant to my situation	“I don’t feel that I need one.”	55 (3.7%)
Time-consuming/too much effort/long wait times	“Would depend if I have the time.”	24 (1.6%)
Preference for alternative treatments	“I would first see a naturopath or seek other alternative medicine paths.”	19 (1.3%)
Personal experience where it wasn’t effective	“I had a laparoscopy last year and will hold off having another one until necessary.”	6 (0.4%)
Would require time off work	“Likely it would require time off work for both the surgery and recovery.”	2 (0.1%)
Neutral intention		
No comment/unclear	“No comment.”	196 (13.3%)
I don’t know/not sure	“I’m not sure.”	49 (3.3%)
Not sure if benefits outweigh cost	“Is it worth the wait time and money—not too sure.”	35 (2.4%)
Depends on how severe and persistent the pain was	“Depends on the severity and how bearable the pain discomfort is.”	22 (1.5%)
Depends if covered by health insurance	“It would depend if it was covered by health insurance.”	6 (0.4%)
Feel neutral	“I feel neutral about it.”	5 (0.3%)

Multiple codes were possible per response and thus may not sum to 100.

### Change in Intention

There was a significant main effect of time, with initial intention to get a laparoscopy (time 1) reduced after being informed of the new endometriosis diagnosis and treatment guidelines (time 2, MD = 0.29, 95% CI = 0.21–0.37, *P* < 0.001, *d* = 0.18; see Table 3). The 2-way interactions between time and post content, *F*(1, 1,469) = 2.23, *P* = 0.14, and between time and source credibility, *F*(1, 1,469) = 2.23, *P* = 0.14, were not significant. The 3-way interaction between time, post content, and source credibility was also not significant, *F*(1, 1,469) = 2.52, *P* = 0.11.

### Attitudes

Controlling for source credibility, attitudes toward laparoscopy were significantly more favorable among those shown the personal anecdote than those shown the nonnarrative content: MD = 0.13, 95% CI = 0.01 to 0.25, *F*(1, 1,469) = 4.68, *P* = 0.03, $\eta_{p}^{2}=\;0.003$ . There was no significant main effect of source, MD = 0.05, 95% CI = −0.07 to 0.17, *F*(1, 1,469) = 0.67, *P* = 0.42, $\eta_{p}^{2}=0.000$ , and the interaction was also not significant, *F*(1, 1,469) = 0.23, *P* = 0.63, $\eta_{p}^{2}=0.000$ .

### Perceived Norms

There were no main effects of content, MD = 0.08, 95% CI = −0.05 to 0.20, *F*(1, 1,469) = 1.49, *P* = 0.22, $\eta_{p}^{2}=\;0.001$ , or source, MD = −0.03, 95% CI = −0.15 to 0.10, *F*(1, 1,469) = 0.16, *P* = 0.69, $\eta_{p}^{2}=\;0.000$ , on perceived norms toward getting a laparoscopy. The interaction was also not significant, *F*(1, 1,469) = 0.06, *P* = 0.81, $\eta_{p}^{2}=0.000$ .

### Self-Efficacy

There were no main effects of content, MD = 0.05, 95% CI = −0.20 to 0.31, *F*(1, 1,469) = 0.18, *P* = 0.67, $\eta_{p}^{2}=0.000$ , or source, MD = −0.15, 95% CI = −0.40 to 0.11, *F*(1, 1,469) = 1.23, *P* = 0.26, $\eta_{p}^{2}=0.001$ , on self-efficacy in getting a laparoscopy for the diagnosis and treatment of endometriosis. The interaction was also not significant, *F*(1, 1,469) = 0.33, *P* = 0.57, $\eta_{p}^{2}=0.000$ .

### Perceived Source Credibility

There was no main effect of content on perceived source credibility, MD = 0.11, 95% CI = −0.01 to 0.23, *F*(1, 1,469) = 3.26, *P* = 0.071, $\eta_{p}^{2}=0.002$ . Perceived source credibility was significantly higher for those shown an Instagram post from an account typically associated with high credibility (“WHO”) than those shown a post from an account typically associated with low credibility (“anonymous user”), MD = 0.29, 95% CI = 0.17 to 0.41, *F*(1, 1,469) = 23.01, *P* = <0.001, $\eta_{p}^{2}=0.015$ . The interaction was not significant, *F*(1, 1,469) = 0.23, *P* = 0.63, $\eta_{p}^{2}=0.000$ .

### Psychosocial Outcomes

Averaged over source, those who viewed the personal anecdote content experienced significantly more positive emotions toward the information in the Instagram post than those who viewed the nonnarrative content, MD = 0.33, 95% CI = 0.18 to 0.48, *F*(1, 1,469) = 18.18, *P* < 0.001, $\eta_{p}^{2}=0.012$ . There was no main effect of source on positive affect, MD = 0.01, 95% CI = −0.15 to 0.16, *F*(1, 1,469) = 0.005, *P* = 0.94, $\eta_{p}^{2}=0.000$ , and the interaction effect was also not significant, *F*(1, 1,469) = 0.14, *P* = 0.71, $\eta_{p}^{2}=0.000$ .

Participants who viewed the nonnarrative content had significantly more negative emotional responses to the information in the Instagram posts compared with those who viewed the personal anecdote, MD = 0.24, 95% CI = 0.09 to 0.40, *F*(1, 1,469) = 9.45, *P* = 0.002, $\eta_{p}^{2}=0.006$ . There was no main effect of source on negative affect, MD = −0.14, 95% CI = −0.29 to 0.02, *F*(1, 1,469) = 2.99, *P* = 0.084, $\eta_{p}^{2}=0.002$ , and the interaction was not significant, *F*(1, 1,469) = 0.86, *P* = 0.36, $\eta_{p}^{2}=0.001$ .

With regard to symptom worry, the main effects of both content, MD = 0.02, 95% CI = −0.06 to 0.12, *F*(1, 1,469) = 0.28, *P* = 0.60, $\eta_{p}^{2}=0.000$ , and source credibility were nonsignificant, MD = −0.76, 95% CI = −0.16 to 0.01, *F*(1, 1,469) = 3.16, *P* = 0.076, $\eta_{p}^{2}=0.002$ ), as was their interaction, *F*(1, 1,469) = 0.73, *P* = 0.39, $\eta_{p}^{2}=0.000$ .

### Knowledge

Controlling for source credibility, post content had no significant effect on knowledge, MD = 0.17, 95% CI = −0.08 to 0.42, *F*(1, 1,469) = 1.83, *P* = 0.18, $\eta_{p}^{2}=0.001$ . After controlling for post content, endometriosis-related knowledge was significantly greater among those shown a post from the low-credibility source than those shown a post from the high-credibility source, MD = 0.27, 95% CI = 0.02 to 0.52, *F*(1, 1,469) = 4.43, *P* = 0.036, $\eta_{p}^{2}=0.003$ . The interaction was not significant, *F*(1, 1,469) = 0.01, *P* = 0.92, $\eta_{p}^{2}=0.000$ .

## Discussion

In this randomized controlled trial of different social media formats on women’s endometriosis treatment-seeking intentions, Instagram posts featuring an anecdote produced higher intentions and more favorable attitudes toward laparoscopy than posts containing nonnarrative content. Taken together with prior research, these results demonstrate the power of personal anecdotes in shaping behavioral intentions as they capture and sustain attention,^29,40^ stimulate an emotional response,^41^ generate personal relevance,^20,42^ and convey the emotional and physical nuances of endometriosis more clearly than scientific information.^3^

A previous study found anecdotes of women’s experiences with endometriosis prompted misinterpretations and misapplications of scientific evidence.^2^ Our findings suggest that using anecdotal formats to share scientific evidence may assist women navigating this complex condition to make informed treatment decisions. In addition, anecdotal stories can stimulate story-consistent attitudinal changes toward public health messages^20^ and beliefs about treatment efficacy.^19^ While hopeful anecdotes have previously predicted positive affect toward pelvic exams and ultrasounds for the diagnosis of endometriosis,^41^ our study found that personal anecdotes can increase positive affect toward the more invasive method of laparoscopy. As many participants mentioned fear of surgery as a barrier to pursuing the procedure in their free-text responses, this is an important finding.

After informing participants that laparoscopy is recommended only when pain medication and hormonal treatments fail to relieve symptoms, intentions decreased across all conditions. Existing literature has found that informing women of the risk of overdetection in mammography screening^27^ and the unreliability of ultrasounds in diagnosing PCOS^28^ led them to reconsider their screening intentions. Within the context of endometriosis, our findings demonstrate that sharing updated evidence and clinical guidelines on social media can promote informed medical decision making, regardless of the type of information that has been previously encountered, or its source. Communicating this to women may reduce the overuse of laparoscopy while also ensuring timely diagnosis.

While the account attributed to the WHO was perceived as more credible, there were no differences in intentions, perceived norms, or self-efficacy toward getting a laparoscopy compared with the layperson account. This contrasts to prior research with university students, in which participants were more likely to act on health information provided by professional Twitter accounts that had a doctor title in their username.^25^ Interestingly, those who saw the post from the low-credibility layperson account had higher knowledge scores than those shown the same information from the high-credibility WHO account, although the overall difference was small. One explanation is that the layperson account may have been perceived as more personally relevant, subsequently increasing attention to the information and facilitating greater retention.^40^ This finding highlights the ability for laypeople to contribute persuasively to endometriosis-related health discussions on social media.

### Strengths and Limitations

To our knowledge, this study was the first in Australia and one of the few worldwide to use participants outside of a university sample to compare the effect of personal anecdotes and nonnarrative social media content on treatment-seeking behavior. Although personal anecdotes account for only approximately 17% of endometriosis-related posts on social media, they tend to yield the highest engagement,^43^ highlighting the practical relevance of our findings.

The randomized design allowed for a robust comparison between groups, with the standardized hypothetical scenarios maximizing internal validity. However, the design was not reflective of the authentic Instagram experience as participants viewed static images of the posts. When engaging with the app directly, women may interact with health information in a more dynamic manner, with the ability to view comments from other users potentially shaping their attitudes toward treatment options. Nonetheless, the controlled nature of the design allowed the effects of the Instagram post manipulations to be directly compared and unbiased by previous experience of an endometriosis diagnosis. While the differences in post content were an intentional part of our experimental design, it remains possible that slight content differences, such as references to variability in symptom experiences and uncertainty regarding causes and treatment, could have influenced treatment-seeking intentions beyond the anecdotal versus nonnarrative format. Therefore, the observed effects may reflect a combination of communication format and informational content. This consideration should be kept in mind when interpreting our findings. It should also be noted that the nonadjustment for multiple comparisons may have resulted in increased type I errors. As this study has established effect sizes that could be anticipated, future studies can be designed that appropriately control the family-wise error rate.

The use of an online panel sample limits our ability to assess whether participants paid attention to the experimental stimuli; however, to mitigate this, we reviewed the free-text responses for coherence and excluded those who completed the survey in less than one-third of the median time. Recruiting participants through a market research company may also introduce selection bias, as these women had preregistered to participate in online surveys and may not be representative of the general population. However more than half of participants had seen a doctor for painful periods, reflecting the demographic often targeted by social media messaging about endometriosis.

The large sample size and quota sampling for high and low education levels offers nuanced insights into how different social media health information formats influence intentions to get a laparoscopy. While statistically significant, these differences between conditions were small. However, given the vast reach of social media and that endometriosis is estimated to affect approximately 190 million individuals globally,^44^ even modest effects of post content and credibility could translate into meaningful, population-level effects on treatment-seeking intentions, warranting further investigation into their clinical relevance.

### Implications

By identifying features of health messages on social media that shape women’s treatment-seeking intentions, attitudes, and knowledge, our research makes meaningful contributions to the development of targeted communication strategies that resonate with those affected by endometriosis. Collaboration among researchers, practitioners, influencers, and social media users is needed to ensure accurate yet engaging health information is shared to online endometriosis communities. Initiatives like the WHO Fides project are working to achieve this by supporting social media influencers in sharing evidence-based messages.^45^ However, more targeted efforts addressing the specific context of endometriosis are essential.

In conclusion, our study showed that formatting health content as a personal anecdote produced higher intentions and more favorable attitudes toward laparoscopy. When advised of new treatment guidelines, intentions reduced, indicating more informed decision making. By providing insights into how women make decisions about their health amidst new evidence and persuasive personal anecdotes on social media, our results highlight the urgent need for effective online health communication strategies. Moving forward, multidisciplinary collaboration on social media is needed to ensure women navigating this complex condition can advocate for appropriate care. In doing so, steps can be made to reduce the diagnostic delay associated with endometriosis, reduce overuse of laparoscopy, and improve women’s health outcomes overall.

## Supplemental Material

sj-docx-1-mdm-10.1177_0272989X261436847 – Supplemental material for Social Media Health Information Formats and Endometriosis Treatment-Seeking Intentions: A Randomized Controlled Trial Supplemental material, sj-docx-1-mdm-10.1177_0272989X261436847 for Social Media Health Information Formats and Endometriosis Treatment-Seeking Intentions: A Randomized Controlled Trial by Alice Graham, Brooke Nickel, Kirsten McCaffery, Jenny Doust, Erin Cvejic and Tessa Copp in Medical Decision Making

sj-docx-2-mdm-10.1177_0272989X261436847 – Supplemental material for Social Media Health Information Formats and Endometriosis Treatment-Seeking Intentions: A Randomized Controlled Trial Supplemental material, sj-docx-2-mdm-10.1177_0272989X261436847 for Social Media Health Information Formats and Endometriosis Treatment-Seeking Intentions: A Randomized Controlled Trial by Alice Graham, Brooke Nickel, Kirsten McCaffery, Jenny Doust, Erin Cvejic and Tessa Copp in Medical Decision Making
