# Supplementary material for: Social Media Health Information Formats and Endometriosis Treatment-Seeking Intentions: A Randomized Controlled Trial
Source: Med Decis Making. 2026 Apr 13;46(6):767–79. doi: 10.1177/0272989X261436847 (PMC13346596; doi:10.1177/0272989X261436847)
Supplement: sj-docx-1-mdm-10.1177_0272989X261436847 – Supplemental material for Social Media Health Information Formats and Endometriosis Treatment-Seeking Intentions: A Randomized Controlled Trial [file sj-docx-1-mdm-10.1177_0272989X261436847.docx]

**Appendix A**

**Instagram Post Stimuli**

*Randomised Group 1*

Instagram post featuring a personal anecdote from a low credibility source.

 
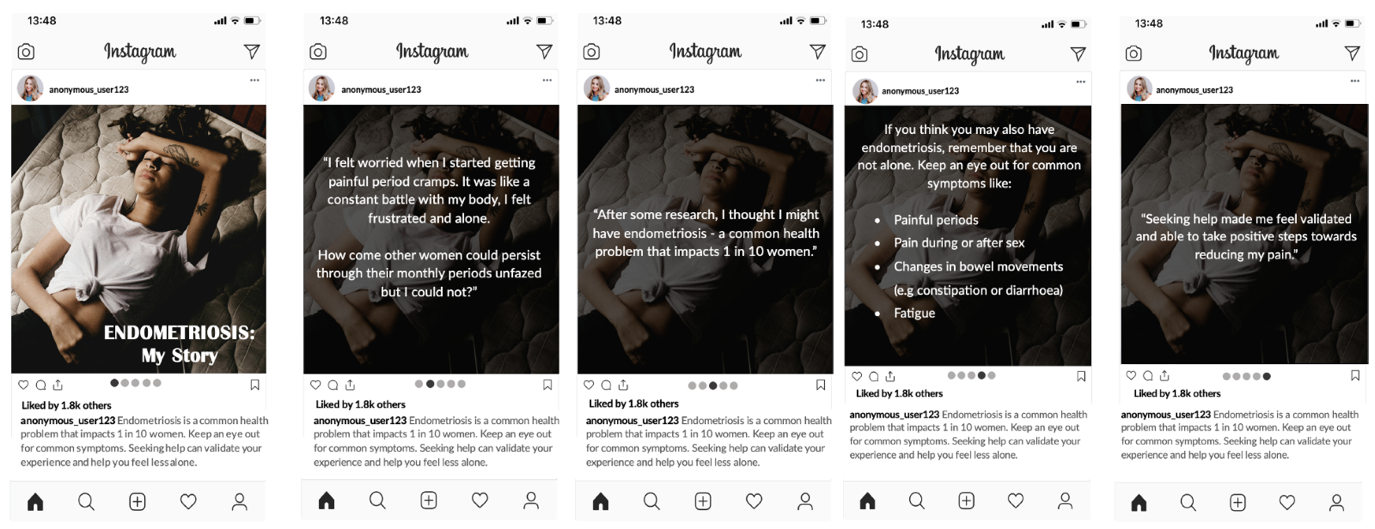


*Randomised Group 2*

Instagram post featuring a personal anecdote from a high credibility source.


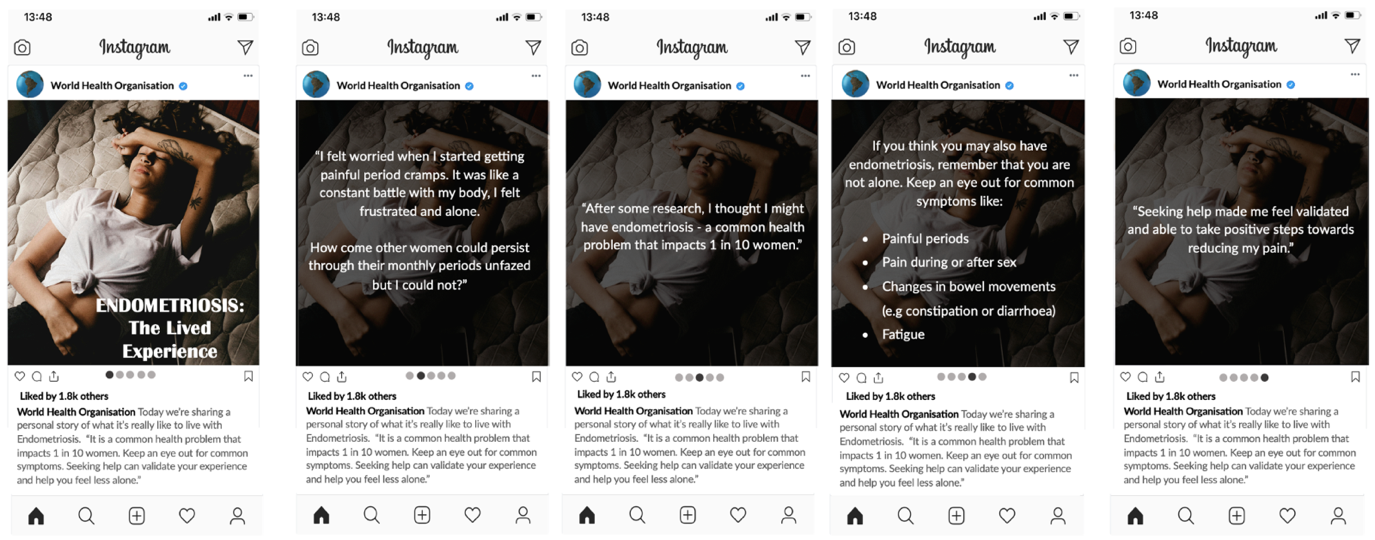


*Randomised Group 3*

Instagram post featuring non-narrative, factual information from a low credibility source.

 
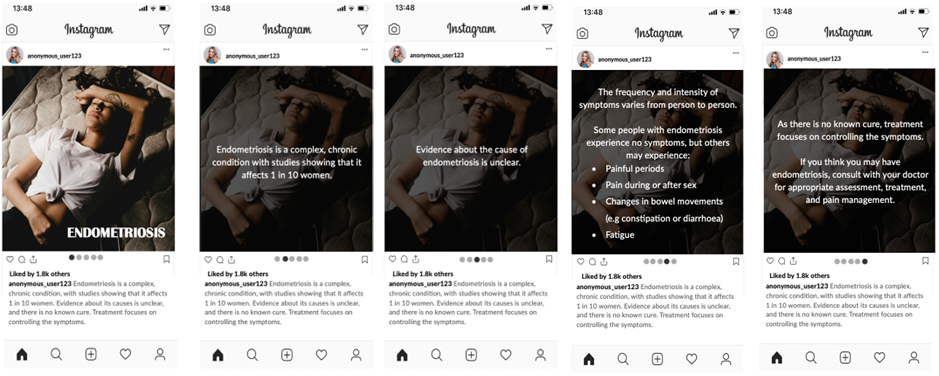


*Randomised Group 4*

 Instagram post featuring non-narrative, factual information from a high credibility source.


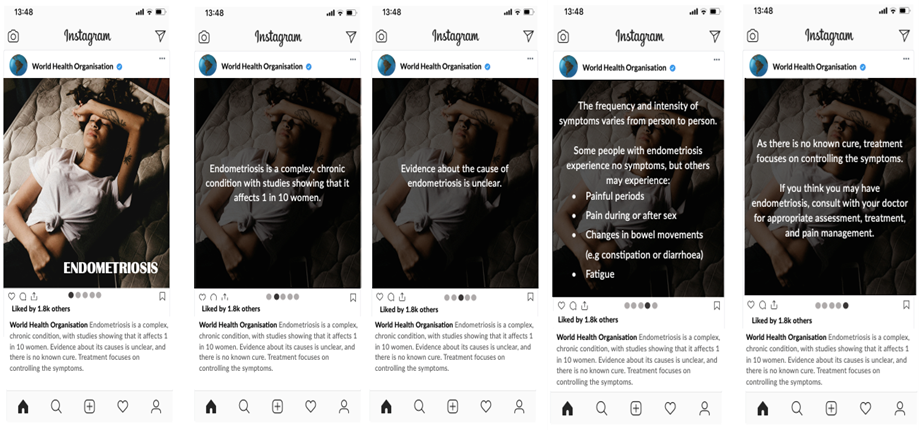


**Appendix B**

**Study Questionnaire**

**[Landing page upon clicking on study link]**

Thank you for your interest in this survey! This study is run by researchers at the University of Sydney and is looking at how social media posts about endometriosis influence diagnosis medical decision making.

To read more about this study, please download the Participant Information Statement

< link to PIS>

I confirm that I have read the Participant Information Statement and consent to take part in this research project as described.

- Yes (*proceed to survey questions on next page*)
- No (*survey ends and no data is collected*)

**[Screeners for eligibility]**

1. How do you describe your gender?
2. Man or male [*direct end of survey*]
3. Woman or female
4. Non-binary or transgender
5. I use a different term (please specify) _____________
6. Prefer not to say
7. At birth, you were recorded as:
8. Male [direct end of survey]
9. Female
10. Another term [direct end of survey]
11. Prefer not to answer [*direct end of survey*]
12. How old are you (in years)? ______ *[<18 or >45 direct to end of survey]*
13. Which country do you live in?
14. Australia
15. Other (please specify) _______ *[direct to end of survey]*
16. Have you ever been personally diagnosed with any of the following diseases?
    1. Endometriosis [*direct end of survey*]
    2. Polycystic Ovary Syndrome
    3. Uterine fibroids
    4. Adenomyosis
    5. Cancer
    6. None

**[Not eligible message:** We’re sorry but you’re not eligible to take this survey. We apologise for any inconvenience this might have caused, and greatly appreciate your interest.**]**

**[Demographics]**

1. Have you ever seen a doctor for painful periods?
   1. Yes
   2. No
2. How often do you use social media for your health?
   1. Daily
   2. Once a week
   3. Once a month
   4. Less than once a month
   5. Never
3. Have you ever experienced difficulties communicating your symptoms to a health care professional in the past?
   1. Yes
   2. No
4. Have you ever felt disbelieved about the severity of your symptoms from a health care professional in the past?
   1. Yes
   2. No
5. [Medical Minimiser Maximiser one-item scale]

Sometimes, medical action is clearly necessary, and sometimes it is clearly NOT necessary. Other times, reasonable people differ in their beliefs about whether medical action is needed. In situations where it’s not clear, do you tend to lean towards taking action or do you prefer to wait and see if action is needed?

Importantly, there is no “right” way to be**.** Please answer on the 1-6 scale below

1. I strongly lean toward waiting and seeing
2. I lean toward waiting and seeing
3. I somewhat lean toward waiting and seeing
4. I somewhat lean toward taking action
5. I lean toward taking action
6. I strongly lean toward taking action
7. What language do you speak at home?
8. English
9. Other (please tell us) ________
10. How well do you speak English?
11. Very well
12. Well
13. Not well
14. Not at all
15. Are you of Aboriginal or Torres Strait Islander origin?
16. Yes
17. No
18. Prefer not to say
19. What is the highest level of education you have completed?
20. Less than Year 12 or equivalent
21. Completed Year 12 or equivalent
22. Trade or technical certificate or diploma
23. University degree
24. Postgraduate/higher degree

The following Instagram post and scenario are about endometriosis. Please read the information very carefully, and keep it in mind when answering the questions that follow.

***[Participants are then randomised to view either:***

1. ***An Instagram post featuring a personal anecdote from a low credibility source.***
2. ***An Instagram post featuring a personal anecdote from a high credibility source.***
3. ***An Instagram post featuring evidence-based information from a low credibility source.***
4. ***An Instagram post featuring evidence-based information from a high credibility source.]***

***[All participants shown scenario of doctor visit]***

Imagine that for the past 12 months; you have had very painful periods. Sometimes, the pain is so intense that you have to call in sick to work. You also feel pain when you go to the bathroom, and during sex. Worried that this is not normal, you make a visit to see your GP.

After checking your symptoms and medical history, the doctor suggests you may have **endometriosis**. This is where uterine tissue grows outside of the womb. They explain that symptoms vary from person to person. You get an ultrasound scan, but the results are unclear. To help with your pain, the doctor suggests taking pain medicine. They also offer to refer you to a gynaecologist, a doctor who specialises in a procedure called laparoscopy.

The doctor explains this would involve having a general anaesthetic (putting you to sleep) while a small cut is made in your stomach. The surgeon would then use a tiny camera to look for excess uterine tissue growing outside of the womb. If found, they will treat it by removing the tissue and send off a sample to confirm the diagnosis. They say that although this is the only way to diagnose endometriosis, the excess tissue can grow back. Also, improvement in pain may only be short-term. They also warn that long wait times are common, and it can cost between $500 - $4000.

Your doctor then asks - would you like to be referred to a gynaecologist for laparoscopy?

**[Post-randomisation outcome measures]**

[Intention to get a laparoscopy]

1. “Which best describes your intentions to get a laparoscopy for diagnosis and surgical treatment of endometriosis, answering as you would in the scenario above?”
   1. (1 = definitely will not, 7 = definitely will).
2. Please tell us why (free text)

[Psychosocial outcomes]

1. How did you feel when you read the information about endometriosis in the Instagram post? (1=not at all to 7= extremely)
   1. Assured
   2. Hopeful
   3. Relieved
   4. Anxious
   5. Afraid
   6. Worried
2. How worried would you feel about your symptoms described in the scenario above if you had them?
   1. 1 = not worried at all
   2. 2 = a bit worried
   3. 3 = quite worried
   4. 4 = very worried

[Attitudes]

1. How beneficial does getting a laparoscopy for the diagnosis of endometriosis seem to you?
   1. 7 point scale: 1=Not at all beneficial to 7= extremely beneficial
2. How harmful does getting a laparoscopy for the diagnosis of endometriosis seem to you?
   1. 7 point scale: 1=Not at all harmful to 7= extremely harmful
3. Do you believe that getting a laparoscopy for the diagnosis of endometriosis is useful?
   1. 7 point scale: 1=not at all to 7= Extremely
4. Do you believe that getting a laparoscopy for the diagnosis of endometriosis is necessary?
   1. 7 point scale: 1=not at all to 7= Extremely

[Perceived Norms]

1. My friends and family would want me to get a laparoscopy for the diagnosis of endometriosis.
   1. 7-point scale: 1 = strongly disagree, 7= strongly agree
2. My friends and family would approve if I got a laparoscopy for the diagnosis of endometriosis.
   1. 7-point scale: 1 = strongly disagree, 7= strongly agree
3. Most women with my symptoms would get a laparoscopy for the diagnosis of endometriosis.
   1. 7-point scale: 1 = strongly disagree, 7= strongly agree

[Attention check]

1. Other than getting a laparoscopy, what was the other option the doctor suggested to manage your symptoms?
   1. Pelvic massage
   2. Acupuncture
   3. Pain medication
   4. Diet Changes

[Self-Efficacy]

1. How confident are you that you can get a laparoscopy for the diagnosis of endometriosis?
   1. 10-point scale: 1 = Not at all confident, 10 = completely confident

[Source Credibility]

1. The Instagram account sharing information about endometriosis is:
   1. 7-point scale: 1 = unreliable, 7 = reliable
2. The Instagram account sharing information about endometriosis is:
   1. 7-point scale: 1 = honest, 7 = dishonest
3. The Instagram account sharing information about endometriosis is:
   1. 7-point scale: 1 = untrustworthy, 7 = trustworthy
4. The Instagram account sharing information about endometriosis is:
   1. 7-point scale: 1 = not an expert, 7 = an expert
5. The Instagram account sharing information about endometriosis is:
   1. 7-point scale: 1 = inexperienced, 7 = experienced
6. The Instagram account sharing information about endometriosis is:
   1. 7-point scale: 1 = unknowledgeable, 7 = knowledgeable

[Knowledge Measures]

1. How many women are affected by endometriosis?
   1. 1 in 10 (correct)
   2. 1 in 5
   3. 1 in 3
   4. 1 in 20
2. Which of the following symptoms are linked with endometriosis? (true/false/DK)
   1. Fever
   2. Painful periods
   3. Headaches
   4. Hair loss
   5. Changes in bowel movements
3. What are the different ways you can treat endometriosis? (true/false/DK)
   1. Antibiotics
   2. Laparoscopy
   3. Pain Medication
   4. Appendectomy

***[Within-Subjects - All participants shown another about new treatment guidelines.]***

Before you leave the appointment, the GP remembers there are new guidelines on Endometriosis. After reading the new guidelines she says that Laparoscopy is now only recommended in patients where pain medicine or hormonal treatment doesn’t work. She also said there is no evidence that an early diagnosis is better than a late diagnosis in preventing the progression of symptoms.

***[Time 2 outcome measure – intention repeated]***

1. “Based on this information from the doctor, which best describes your intentions to get a laparoscopy for diagnosis and surgical treatment of endometriosis?”
   1. (1 = definitely will not, 7 = definitely will).
2. Please tell us why (free text)

Thank you for taking part in this study! Please access these resources about endometriosis if you would like more information:

- Endometriosis Australia: <https://endometriosisaustralia.org/?gad_source=1&gclid=Cj0KCQjwiYOxBhC5ARIsAIvdH51N7WXEzxGold1McCj4bxfw-1A_6kjg8e5HTcGt7uokoctpo1FII8EaAkBrEALw_wcB>
- Endometriosis and pelvic pain clinics: <https://www.health.gov.au/our-work/endometriosis-and-pelvic-pain-clinics#current-endometriosis-and-pelvic-pain-clinics>
- Symptom Checker: <https://www.endozone.com.au/health-report-form#no-back>
