# Supplementary material for: Social Media Health Information Formats and Endometriosis Treatment-Seeking Intentions: A Randomized Controlled Trial
Source: Med Decis Making. 2026 Apr 13;46(6):767–79. doi: 10.1177/0272989X261436847 (PMC13346596; doi:10.1177/0272989X261436847)
Supplement: sj-docx-2-mdm-10.1177_0272989X261436847 – Supplemental material for Social Media Health Information Formats and Endometriosis Treatment-Seeking Intentions: A Randomized Controlled Trial [file sj-docx-2-mdm-10.1177_0272989X261436847.docx]

**Supplemental Materials**

**Outcome Measures**

**Psychosocial Outcomes.** Two scales were used to assess participants’ psychosocial outcomes. The first was an adapted version of the Berlin Emotional Responses to Risk Instrument (BERRI)^Petrova, Cokely 1^ which included six items on a 7-point scale (1= *Not at all* to 7= *Extremely*) assessing participants emotional responses to the risk information in the Instagram posts. e.g., “*how hopeful did you feel when you read the information about endometriosis in the Instagram post?”.* Scale scores were created by averaging scores across the measures of negative affect (BERRI-neg) and the measures of positive affect (BERRI-pos), with higher scores indicating a stronger emotional reaction to risk information.

The second was an adapted^2^ single item measuring symptom worry [*“How worried would you feel about your symptoms described in the scenario above if you had them?*”] on a 4-point scale (1 = *Not at all worried* to 4 = *Very worried*).

**Attitudes**. Attitudes towards getting a laparoscopy were measured using an adapted scale^3^ consisting of four items each measured on a 7-point scale (1= *Not at all* to 7= *Extremely*) e.g., “*How beneficial does getting a laparoscopy for the diagnosis of endometriosis seem to you?*”. Relevant items were reverse-coded, and items were averaged to create a composite score. Higher scores indicated a more favourable attitude toward laparoscopy.

**Perceived Norms.** Perceived norms towards getting a laparoscopy were measured using an adapted scale^3^ which included 3 items measured on a 7-point scale (1= *Strongly disagree* to 7= *Strongly agree*) e.g., “*My friends and family would approve if I got a laparoscopy for the diagnosis of endometriosis if I had these symptoms.*” Items were averaged to create a composite score with higher scores indicating a stronger perception of normative support for laparoscopy.

**Self-Efficacy.** A single adapted item^4^ was used to measure perceived self-efficacy about getting a laparoscopy (e.g., *How confident are you that you can get a laparoscopy for the diagnosis of endometriosis?*) on a 10-point scale (1 = *Not at all confident* to 10 = *Completely confident*).

**Perceived Source Credibility.** The perceived source credibility of the Instagram account was measured using an adapted scale^5^ consisting of six items measured on a 7-point scale, e.g., [“*The Instagram account sharing information about endometriosis is:”* 1 = *Untrustworthy* to 7 = *Trustworthy*]. Items were averaged to create a composite score with higher scores indicating higher perceived credibility.

**Knowledge Measures.** Ten items were included to assess participants’ knowledge of endometriosis (e.g., “*how many women are affected by endometriosis?*”) based on information that was presented in the Instagram posts and hypothetical scenario. Responses were categorised as either 0 = *Incorrect* or 1 = *Correct*). An overall score was computed by summing the items, with higher scores reflecting greater knowledge.

1. Petrova D, Cokely ET, Sobkow A, Traczyk J, Garrido D, Garcia‐Retamero R. Measuring feelings about choices and risks: The Berlin Emotional Responses to Risk Instrument (BERRI). Risk analysis. 2023;43(4):724-46.

2. Sutton S, Bickler G, Sancho-Aldridge J, Saidi G. Prospective study of predictors of attendance for breast screening in inner London. Journal of epidemiology and community health (1979). 1994;48(1):65-73.

3. Wong P, Ng PML, Lee D, Lam R. Examining the impact of perceived source credibility on attitudes and intentions towards taking advice from others on university choice. International journal of educational management. 2020;34(4):709-24.

4. Schwarzer R, Jerusalem M, Weinman J, Wright S, Johnston M. Generalized Self-Efficacy Scale. Measures in Health Psychology: A User's Portfolio Causal and control beliefs Windsor. 1995.

5. Lee JY, Sundar SS. To Tweet or to Retweet? That Is the Question for Health Professionals on Twitter. Health communication. 2013;28(5):509-24.
